# 异种移植抗原α-gal介导人血清杀伤A549细胞的实验研究

**DOI:** 10.3779/j.issn.1009-3419.2012.11.05

**Published:** 2012-11-20

**Authors:** 圣明 朱, 玲 谢, 鸿 郑, 凤 秦, 美 刘, 志国 骆, 艳萍 王

**Affiliations:** 1 442000 十堰，湖北医药学院附属太和医院肿瘤科 Department of Oncology, Taihe Hospital, Affiliated to Hubei Medical College, Shiyan 442000, China; 2 442000 十堰，湖北医药学院附属太和医院皮肤科 Department of Dermatology, Taihe Hospital, Affiliated to Hubei Medical College, Shiyan 442000, China; 3 610041 成都，四川大学华西医院肿瘤分子诊断研究室 The Laboratory of Tumor Molecular Diagnosis, West China Hospital, Sichuan University, Chengdu 610041, China

**Keywords:** *α1, 3-GT*基因, α-半乳糖基, 异种抗原, A549细胞, α-1, 3-Galactosyltransferase, α-Galactosyl, Xenoantigen, Human adenocarcinoma cell A549

## Abstract

**背景与目的:**

人体α-1, 3半乳糖基转移酶（α-1, 3Galactosyltransferase, α-1, 3GT）基因功能失活而不表达α-半乳糖基（α-galactosyl, α-gal）表位，但天然存在着大量抗α-gal抗体，异种器官移植研究结果提示，在人肿瘤细胞上重新表达异种移植抗原α-gal，可能诱发类似于宿主抗移植物超急性排斥反应的抗肿瘤效应。本研究通过基因导入手段，建立稳定表达异种移植抗原α-gal的转基因人肺癌细胞系，探讨α-gal介导的人血清抗肿瘤的可能性及其机制。

**方法:**

将前期成功构建的*α-1, 3GT*基因真核表达质粒pEGFP-N1-GT瞬时转染人肺腺癌细胞A549，筛选并建立稳定的转基因细胞系A549-GT。MTT增殖实验和显微镜观察转基因细胞生物学特性变化；RT-PCR检测A549-GT中*α-1, 3GT*基因mRNA表达；荧光素标记的凝集素（FITC-BS-IB4 lectin）染色检测α-1, 3GT在肿瘤细胞表面合成α-gal的能力；A549-GT细胞及其培养基分别与正常人细胞共培养，检验*α-1, 3GT*基因的稳定性和酶活性的稳定性；人血清结合实验检测A549-GT与IgM和补体C3结合情况。

**结果:**

RT-PCR检测到转基因细胞系A549-GT中有α-1, 3GT mRNA表达。荧光显微镜和流式细胞术检测结果显示：A549-GT能够长期稳定表达异种移植抗原α-gal表位；A549-GT与其亲本细胞在生长形态及增殖速度上无明显差异；A549-GT细胞及其培养基分别与正常人胚肺成纤维细胞MRC-5共培养均不能使MRC-5获得α-gal合成能力；经人血清处理后，荧光免疫实验观察到转基因细胞系A549-GT能与血清抗体IgM结合并诱导补体C3结合。

**结论:**

异种移植抗原α-gal在肿瘤细胞上的重新表达，可能通过补体依赖的细胞毒机制，介导类似于异种器官移植排斥反应的抗肿瘤效应。

## Abstract (en)

**Background and objective:**

The absence of α-gal in humans is caused by the inactivity of *α-1, 3GT* gene. However, humans have pre-existing and abundant anti-gal antibodies. Xenotransplantation procedures have indicated the high potential of introducing *α-1, 3GT* gene to synthesize α-gal for cancer gene therapy by mimicking hyper-acute rejection. The aim of this study is to construct a lung cancer A549 cell line that expressed α-gal, and to observe the antitumor mechanisms mediated by human serum.

**Methods:**

A549 cells were transfected with pEGFP-N1-GT plasmids constructed in a previous study. A stable transgenic cell line, A549-GT, was then selected and cultivated. The biological characteristics of A549-GT cells, including morphology and proliferation, were examined. α-1, 3GT mRNA expression was detected by RT-PCR. Direct immunofluorescence staining and flow cytometry (FCM) were used to analyze the synthesis of α-gal in A549-GT. The binding of human serum IgM and C3 with A549-GT were also detected.

**Results:**

α-1, 3GT mRNA was expressed in A549-GT. Direct immunofluorescence staining and FCM indicated a high and stable α-gal expression rate in A549-GT. Compared with parental A549 cells, the biological characteristics of A549-GT were unaltered. α-Gal expression was not detected in the human fetal lung fibroblast cell line MRC-5 even though A549-GT and its culture medium were cultivated with the enzyme. Immunofluorescence staining and FCM also indicated abundant binding between A549-GT treated with human serum and IgM/C3.

**Conclusion:**

α-Gal expression in tumor cells by gene transduction can induce complement-dependent cytototic antitumor effects.

近年来，以利妥昔单抗和曲妥珠单抗为代表的治疗性单抗药物的开发和应用成为肿瘤分子靶向治疗领域最为成功的代表，该类药物以肿瘤相关抗原（tumor associated antigen, TAA）作为分子靶标，通过与抗原结合，介导补体依赖的细胞毒性作用、抗体依赖的细胞毒性等多种机制杀伤肿瘤细胞^[1-3]^。然而，至今正式应用于临床治疗的治疗性单抗药物多集中在血液系统肿瘤、乳腺癌和大肠癌等少数肿瘤。其重要原因在于选择合适的靶抗原是成功设计与开发肿瘤治疗性单抗的前提和关键，而目前除了少数肿瘤之外，大部分肿瘤尤其是高复发率肿瘤的TAA很难明确，即便是在TAA较清楚的少数肿瘤，由于肿瘤细胞间存在的异质性和肿瘤患者个体差异性使得针对TAA单抗药物的开发和应用受到了限制^[4]^。随着分子生物学技术的飞速发展，一些肿瘤研究者开始尝试通过基因治疗手段，重新在人肿瘤细胞上合成人类不表达的异种抗原，利用人体天然存在的免疫体系，诱导了一系列有效的抗肿瘤效应，显示出良好的应用前景。

α-gal是广泛存在于猪、牛、鼠等非灵长类哺乳动物体内的一类碳水化合物表位。在物种进化的过程中，由于负责催化合成α-gal的半乳糖基转移酶基因的移码突变和无意义突变，人体不能合成α-gal，但人体天然预存着大量针对α-gal的抗体，这些抗体能特异性识别并结合外源性α-gal表位。移植免疫学研究证实^[5]^，诱发猪-人器官移植过程中超急性免疫排斥反应的关键因素正是在于，表达在猪血管内皮细胞上的α-gal能同人体天然存在的α-gal抗体迅速结合，通过CDC途径引起移植物细胞的溶解和血管内皮细胞的损伤。这提示，利用α-1, 3GT的基因导入，在肿瘤细胞上重新合成α-gal，构建能被人体天然抗体识别且特异性结合的异种靶标，有可能介导类似于异种移植排斥反应的抗肿瘤效应。本研究通过转基因手段建立稳定表达异种移植抗原α-gal的人肺癌细胞系，探讨α-gal诱导的人血清抗体/补体系统免疫激活机制，以期为肿瘤生物治疗提供新思路。

## 1 材料与方法

### 1.1 实验材料

人肺癌细胞系A549、人胚肺成纤维细胞MRC-5和已知α-gal阳性表达的猪髂动脉内皮细胞PIEC均由四川大学华西医院肿瘤分子诊断实验室提供，用含有15%新生小牛血清的DMEM培养基培养。pEGFP-N1-GT^[6]^是CMV启动子调控的*α-1, 3GT*基因表达质粒为本课题组在前期工作中构建。DMEM培养基、脂质体转染试剂盒lipofectamine™2000、Trizol总RNA提取试剂盒购自Invitogen公司。荧光素标记的植物凝集素（FITC-BS-IB4）为Vector公司产品。M-MLV逆转录酶、新霉素衍生物G418购自美国Promega公司。高保真DNA聚合酶KOD-Plus DNA polymerase购自日本Toyobo公司。MTT购自美国Sigma公司。荧光素标记的羊抗人IgM（FITC-anti-IgM）及羊抗人C3（FITC-anti-C3）均购自北京中杉金桥公司。健康人混合型血清为四川大学华西医院中心血库提供。

### 1.2 实验方法

#### 1.2.1 细胞培养

37 ℃、5%CO_2_条件下，用含10%小牛血清的DMEM常规培养人肺腺癌细胞A549、MRC-5和PIEC细胞。

#### 1.2.2 质粒转染和稳定转染细胞株的筛选

将对数生长期细胞A549细胞接种于6孔板中，24 h后细胞长至80%-90%时，按照lipofectamine™ 2000说明书将pEGFP-N1-GT转染A549细胞。48 h后，将转染细胞以1:5传代，同时换用400 mg/mL的G418细胞筛选培养基，G418的浓度隔日增加100 mg/mL，维持G418浓度为800 mg/mL至3周后形成单克隆；共挑取10个克隆扩大培养，并间歇给予400 mg/mL的G418，在此期间，每月利用直接荧光免疫染色法检测细胞表面α-gal的表达，经筛选得到的稳定表达α-gal的A549细胞命名为A549-GT。下述实验均采用转染24个月后的A549-GT细胞，并将A549细胞作为其母本对照组，将无启动子的空质粒转染细胞A549-p1-GT，作为平行对照组。

#### 1.2.3 *α-1, 3GT*基因mRNA的表达检测

按照Trizol总RNA提取操作方法，提取转染细胞总RNA。以总RNA为模板，利用RT-PCR的方法检测上述细胞中α-1, 3GT mRNA的表达。根据GenBank中猪α1, 3-GT的序列（NM_213810）及内对照（人GAPDH）的序列设计引物，F1：5’-TCAATGCTGCTTGTCTCA -3，R1：5’-TAAGTGCCTTCCCATA-3’（扩增300 bp的α-1, 3GT片段）；F2：5’-GTCAGTGGTGGACCTGACCT-3’，R2：5’-AGGGGAGATTCAGTGTGGTG-3’（扩增395 bp的GAPDH片段）逆转录后，PCR条件为94 ℃预变性2 min，94 ℃ 30 s，50 ℃ 30 s，72 ℃ 2 min，33个循环，72 ℃后延伸10 min。RT-PCR产物用15 g/L琼脂糖凝胶电泳检测。

#### 1.2.4 A549-GT细胞上α-gal抗原表位的表达检测

植物凝集素（BS-IB4 lectin）能与α-gal特异性结合，故本实验采用荧光素标记的凝集素（FITC-BS-IB4 lectin）检测96孔板转染细胞中α-gal的表达。在96孔板中接种细胞，用DMEM培养液以1:50稀释BS-IB4 lectin，向每孔加入50 μL稀释液，室温避光20 min。在荧光倒置显微镜下每月1次检测细胞上α-gal的表达情况。流式细胞仪检测：另取一定数量细胞分至EP管中，1%BSA洗细胞3次；每4×10^5^细胞加入含8 μg BS-IB_4_ lectin的1%BSA液300 μL，4 ℃避光1.5 h；1%多聚甲醛固定，4 ℃避光30 min；1%BSA洗3次；加入300 μL 1%BSA，重悬，上机检测，以A549细胞为阴性对照，每组重复3次。

#### 1.2.5 α-gal在A549-GT细胞膜上的稳定性检测

取6孔板，在同一孔中接种10^5^个A549-GT和10^5^个MRC-5细胞，常规培养24 h；另取一孔，接种10^5^个MRC-5细胞，12 h后，PBS清洗细胞，将培养瓶中A549-GT细胞培养液稍加离心后，上清加入MRC-5细胞中，继续培养24 h。按1.2.4方法，荧光显微镜观察细胞表面α-gal的表达情况。

#### 1.2.6 A549-GT与血清IgM和血清补体C3结合分析

##### 1.2.6.1 荧光显微镜下观察转染细胞与人血清中IgM的结合情况

在96孔板中接种细胞，PBS洗3次。以下过程均在4 ℃下进行。2%多聚甲醛固定10 min。PBS洗3次。每孔加入人血清100 μL，4 ℃静置1 h。吸去血清，PBS洗3次。每孔加入50 μL用DMEM液按1:50稀释的羊抗人FITC-anti-IgM，静置30 min，PBS洗3次。以A549细胞为阴性对照，荧光倒置显微镜下观察人血清中IgM与细胞的结合情况。

##### 1.2.6.2 流式细胞仪检测转染细胞与人血清中IgM的结合情况

消化细胞分至EP管中，1%多聚甲醛4 ℃固定细胞30 min；1%BSA洗2次；加入用DMEM稀释的80%的人血清300 μL，悬浮细胞，37 ℃，1 h；1%BSA洗3次；用DMEM按1:50稀释羊抗人FITC-anti-IgM，按每1×10^6^细胞加入稀释液200 μL，4 ℃，避光静置30 min。1%BSA洗3次；加入300 μL 1%BSA，重悬细胞，上机检测，以A549细胞为阴性对照，重复3次。

##### 1.2.6.3

分别参照步骤1.6.1和1.6.2中转染细胞与血清IgM结合的分析方法，将羊抗人FITC-IgM改为羊抗人FITC-anti-C3，利用荧光显微镜和流式细胞仪检测转染细胞与血清补体C3的结合情况。

#### 1.2.7 细胞生长曲线和细胞形态观察

按1×10^4^个/孔在96孔板上接种A549、A549-G、A549-p1-GT细胞，每种细胞设置5个复孔。5%CO_2_、37 ℃培养。分别在细胞接种24 h、48 h、72 h、96 h、120 h、144 h后取上述96孔板中细胞，每孔加入10 μL MTT（5 mg/mL），37 ℃继续培养4 h；去上清后每孔加入DMSO150 μL，570 nm波长检测OD值；统计学处理所测得数据。以时间为横坐标，OD值为纵坐标，绘制生长曲线。培养过程中，光镜下观察A549、A549-G细胞的形态。

### 1.3 统计学分析

采用SPSS 11.0进行统计学分析，以*P* < 0.05为有统计学意义。

## 2 结果

### 2.1 A549-GT中α-1, 3GT mRNA的表达

RT-PCR琼脂糖凝胶电泳分析显示：转基因细胞系A549-GT细胞中有α-1, 3GT mRNA的表达，而A549细胞及空质粒转染细胞A549-p1-GT则无（图 1），细胞传代24个月后重复实验仍能得到同样的结果。

**1:**
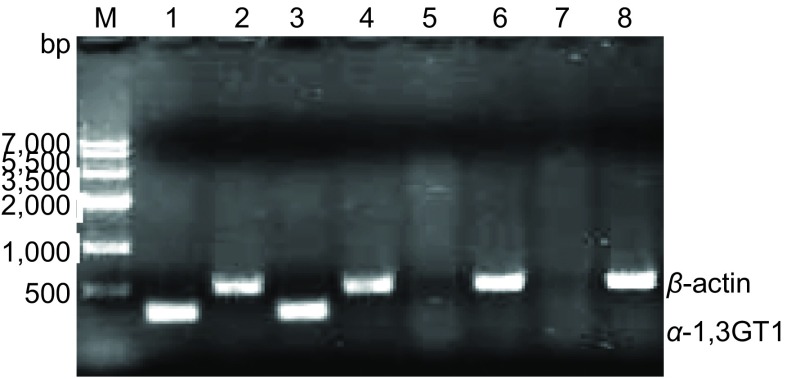
A549-GT细胞中*α*-1, 3GT mRNA的表达 The expression of *α*-1, 3GT mRNA in A549-GT cells. M: marker; 1-4: A549-GT; 5, 6: A549; 7, 8: A549-p1-GT.

### 2.2 A549-GT细胞中α-gal的表达

直接免疫荧光染色法观察结果显示：A549-GT和PIEC细胞中有较强的荧光，A549细胞中无荧光（图 2）；FCM分析显示：稳定转染24个月后A549-GT阳性表达细胞达（80.1±3.2）%，阳性对照细胞PIEC上α-gal的表达率达80%，对照组母本细胞A549的阳性细胞百分比仅（1.1±0.8）%。

**2:**
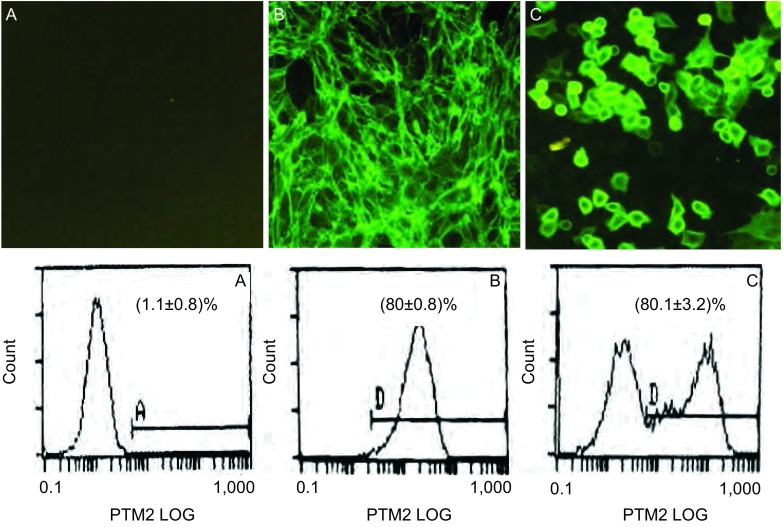
A549-GT细胞中*α*-gal表达免疫荧光观察（×200）及流式细胞分析 Immunofluorescence observation and FCM analysis of the expression of *α*-gal on A549-GT. A: A549; B: PIEC; C: A549-GT.

### 2.3 A549-GT的细胞生物学特性

在常规培养上述细胞的过程中，显微镜下观察到：稳定表达α-gal的A549-GT细胞与A549细胞之间细胞形态没有明显差异。MTT检测稳定表达α-gal的A549-GT细胞生长情况，生长曲线结果（图 3）显示，A549细胞及空质粒转染细胞A549-p1-GT和A549-GT细胞增殖速度无统计学意义（*P* > 0.05）。

**3:**
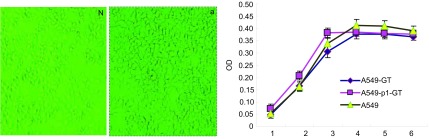
*α*-gal的表达对A549转基因细胞形态（×100）及增殖的影响 The effects of *α*-gal on the morphology and proliferation of A549 transfected cells. N: A549-GT; a: A549.

### 2.4 α-1, 3GT对周围正常细胞的影响

A549-GT细胞与MRC-5共培养以及A549-GT培养基与MRC-5共培养24 h后，植物凝集素的直接免疫荧光结果显示：两种培养方式均只在A549-GT细胞上观察到荧光，MRC-5细胞无荧光（图 4），说明α-1, 3GT牢固地锚定在转基因细胞内，对周围正常细胞未产生“旁观者”效应。

**4:**
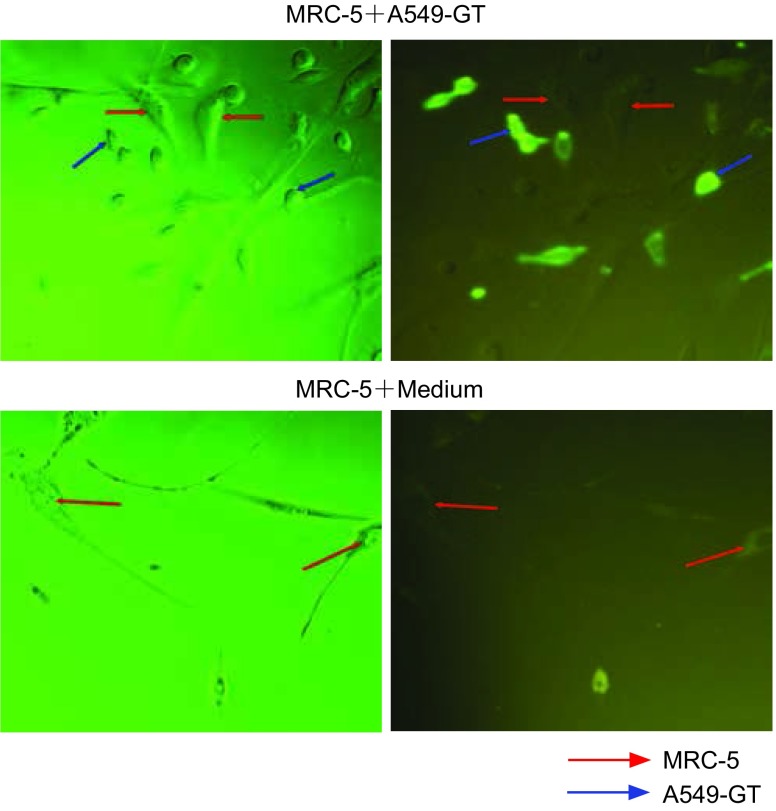
*α*-1, 3GT对周围正常细胞的影响 The effect of *α*-1, 3GT on normal cell

### 2.5 A549-GT细胞与血清IgM和血清补体C3的结合

细胞经健康人血清处理，再与FITC-anti-IgM共同孵育后，荧光倒置显微镜下观察到：表达α-gal的A549-GT细胞膜上有大量的荧光，而不表达α-gal的A549细胞膜上则仅见本底性结合（图 5）。FCM分析结果显示：A549和A549-GT经血清处理后细胞膜上检测到的平均荧光强度分别为4.68±0.22、45.9±0.46。细胞经人血清处理，与FITC-anti-C3共同孵育后，荧光倒置显微镜下观察到：A549-GT细胞膜上有大量FITC-anti-C3的沉积，而A549细胞膜上则仅见本底性结合（图 5）。FCM分析结果显示：A549和A549-GT经血清处理后细胞膜上检测到的平均荧光强度分别为4.5±0.17、37.5±0.36。

**5:**
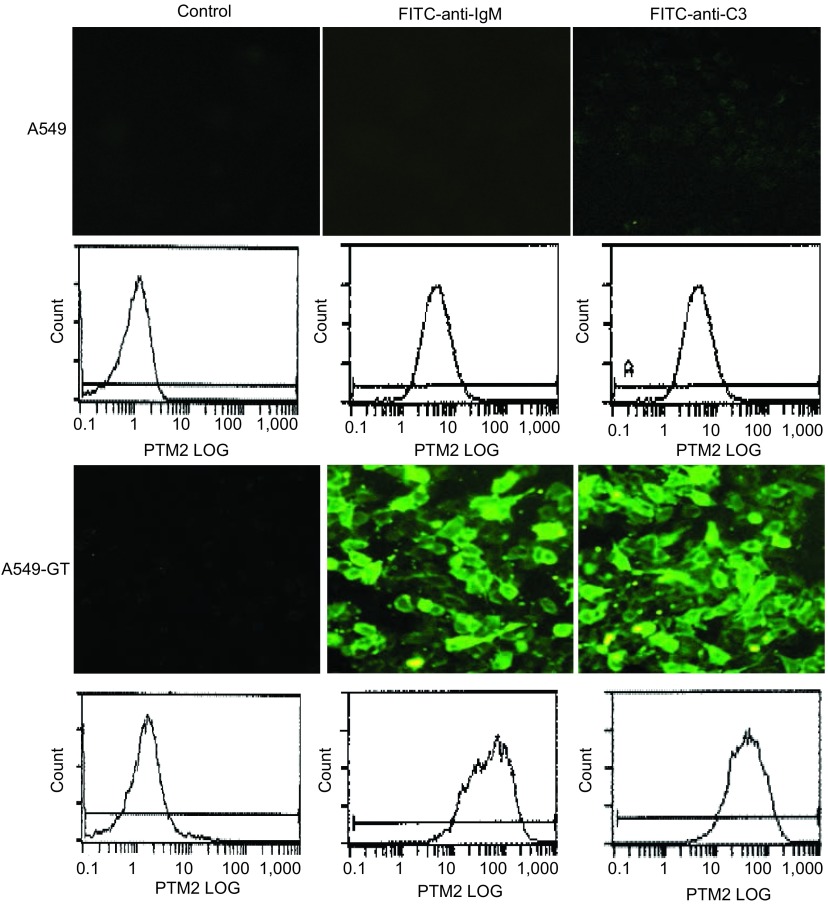
荧光显微镜观察及流式细胞分析A549-GT细胞与血清中IgM/C3的结合 The binding of A549-GT on IgM/C3 by fluorescence microscope and FCM

## 3 讨论

α-gal（Galα-1, 3Gal-β1-4-GlcNAc-R）是细胞表面糖蛋白和糖酯上Galα(1-3)Gal双糖末端残基结构，广泛表达于除人、猿和古世纪候之外的哺乳动物细胞表面，是由α-1, 3半乳糖基转移酶（α-1, 3GT）负责催化合成。在生物进化过程中，由于基因突变，α-1, 3GT功能失活，不能合成α-gal。Lanteri等^[7]^克隆了人*α-1, 3GT*基因并与小鼠*α-1, 3GT*基因做比对，结果证实，两者在基因结构上具有相似性，但由于强转录终止信号的提前出现，使得人α-1, 3GT mRNA只转录出4个截短的外显子区，而缺失了两个具有催化功能的外显子区，这导致了人截短的、无功能的*α-1, 3GT*假基因的形成。本研究，将前期建成功的CMV启动子调控的α-1, 3GT基因真核表达载体pEGFP-N1-GT转染A549后，RT-PCR显示正常人肺腺癌细胞A549中无*α-1, 3GT*基因表达，而转基因细胞A549-GT中有大量α-1, 3GT mRNA转录产物。植物凝集素（BS-IB4）具有与α-gal表位特异结合的特点，植物凝集素标记下的直接免疫荧光染色法和流式细胞术均检测到A549-GT细胞中有丰富的α-gal合成，而A549中无α-gal合成，这提示人体因*α-1, 3GT*假基因的存在不表达α-gal，但有可能利用基因导入的方法，将外源性*α-1, 3GT*基因导入人肿瘤细胞，重新在肿瘤细胞上合成α-gal。国内外也有一些研究者借助基因重组技术，首先通过昆虫、病毒和细菌等生物容器中获取a-1, 3GT，再用神经氨酸酶处理肿瘤细胞，最后与尿苷二磷酸半乳糖（UDP-Gal）共孵，在肿瘤细胞表面合成α-gal表位，但这些方法不仅步骤繁琐、产量不高，且在人体应用过程中，残留的病毒蛋白和细菌内毒素可能给患者带来不必要的损伤^[8-10]^。

为探讨α-gal表位在肿瘤细胞中的重新合成对肿瘤细胞生物学特性的影响，本研究通过MTT增殖实验比较了转基因细胞A549-GT及其亲本细胞A549的增殖活性，结果未发现明显差别。与本研究结果一致的是，Xing等^[11]^发现在胃癌细胞、黑色素瘤细胞和人肺癌细胞上合成α-gal改变了细胞表面能与VVA，PNA等相互作用的碳链结构，而肿瘤细胞的生长速度并没有受到明显的影响。Aubert等^[12]^报道，表达α-gal的胰腺癌细胞Bx-αGT.9和Pc-αGT.2与其母本细胞的生长速度没有明显差别，但却修饰了细胞表面碳链结构，改变了细胞表面其它与肿瘤细胞生物学特性相关的碳水化合物残基结构，可能会改变胰腺癌症细胞之间及同周围基质的粘附能力，甚至降低胰腺癌细胞的转移能力。随后，研究者利用金仓鼠细胞系HaP-T1做了类似的研究，结果得到相同的结论^[13]^。

α-1, 3半乳糖苷基转移酶位于细胞高尔基体，为一典型的Ⅱ型跨膜蛋白，包含一短的胞质尾区、一跨膜区、一主干区和一个羧基末端催化区，有学者以可溶形式表达了α-1, 3GT截短的催化结构域，同样可以在肿瘤细胞表面合成α-gal表位^[10]^，但若将这种可溶的α-1, 3GT用于人肿瘤治疗，则可能使得周围的正常细胞产生“旁观者”效应，本研究将α-1, 3GT全长基因克隆至表达载体，不仅能在肿瘤细胞上长期稳定合成α-gal，而且转基因细胞及其培养基分别与正常人胚肺成纤维细胞MRC-5共培养，均不能使MRC-5获得α-gal合成能力，这说明α-1, 3GT在转基因细胞内的锚定具有牢固性，不会轻易脱落“感染”周围正常组织，增加了其应用于人体时的靶向性。此外，本研究发现，α-1, 3GT酶活性稳定，转基因细胞24个月后仍能大量合成α-gal表位，且α-gal未被人体其它酶类消化掉，保证了其稳定性和有效性。

虽然人体不能合成α-gal表位，但正如远古时代人类祖先能对表达α-gal的感染性微生物产生防御一样，人类对α-gal并不耐受，作为对栖身于胃肠道且表达α-gal的微生物的免疫应答，正常人从婴儿开始，由占循环总量1%的B淋巴细胞产生大量针对α-gal的天然抗体（anti-gal），包括IgG、IgM和IgA^[14]^。已有的研究^[15]^表明：由于α-gal表位缺乏唾液酸且完全没有静电电荷，anti-gal抗体对α-gal的结合具有非常高的特异性，它只能结合到细胞表面糖脂的α-gal残基上，而不能结合到哺乳动物细胞表面其它碳水化合物表位上^[16]^。异种器官移植免疫研究显示，导致移植物超急期排斥反应的主要机制是人血清中抗α-gal抗体与猪组织细胞上α-gal特异性结合，激活了补体系统，在移植物细胞膜上形成膜攻击复合体，裂解表达α-gal的猪组织细胞，导致器官移植失败。为此，国内外一些研究者正积极寻求克服这种排斥反应的有效手段，如培育*α-1, 3GT*基因敲除猪等^[17]^。本研究反向思维，通过基因导入手段在人肿瘤细胞上重新合成了α-gal表位，以期模拟HAR机制发挥抗肿瘤作用。在本实验中，分别向A549-GT和A549细胞中加入正常人血清，再与荧光素标记的抗IgM抗体（FITC-anti-IgM）共孵育后，荧光显微镜及流式细胞术均检测到转基因细胞A549-GT细胞膜上有大量anti-gal抗体结合，而A549细胞膜上则无结合，且人血清天然抗体对肿瘤细胞的结合依赖于肿瘤细胞上α-gal的表达。

Aubert等^[13]^发现，表达a-gal的Bx-aGT9和PC-aGT2细胞不仅能引起anti-gal抗体与之结合，还能进一步诱导与之结合的anti-gal抗体上补体因子的大量结合。为进一步证实表达a-gal的肺癌细胞与血清抗体结合后能否激活补体系统，本研究将A549-GT细胞和正常人血清共同孵育，荧光素标记的补体结合实验显示：稳定表达α-gal的A549-GT细胞膜上有大量补体C3的结合，而不表达α-gal的A549细胞膜上则未见荧光。这提示，利用基因转导手段向人肺癌细胞中导入功能性*α-1, 3GT*基因，为人血清中天然抗体攻击肿瘤细胞构建特异性靶标，可能诱导血清抗体的结合及补体的激活，进而发挥抗肿瘤效应。Unfer的研究小组^[18]^将表达a-gal的MC38细胞与人血清共同孵育，补体依赖的细胞毒杀伤实验显示：与50%人血清共同孵育的表达a-gal的MC38细胞有98%被裂解。Yoshimura等^[19]^在肝癌和胰腺癌细胞上观察到同样的现象。郭军^[20]^的研究报道，将稳定表达α-gal的胃癌细胞株GC9811与不同浓度的人血清孵育后，观察到GC9811细胞能被不同浓度的血清有效的裂解，且裂解作用具有血清浓度依赖性。

和其它肿瘤自杀基因、凋亡基因用于肿瘤基因治疗一样，将*α-1, 3GT*基因导入肿瘤细胞中也需面临靶向性问题，假设α-gal抗原在肿瘤之外的正常组织中表达，不仅会产生类似异种器官移植的强烈排斥反应，使正常组织受到损伤，还会增强正常人体细胞的免疫原性，引起严重的自身免疫性疾病，如Graves’病等^[21, 22]^。因此，积极探索靶向性更强的基因表达方式是开发α-gal用于肿瘤基因治疗必须解决的难题之一。但国内外的大多数研究者似乎并没有对这种严重后果给予足够的重视。Lanteri的课题组将长约250 bp的*neu*基因启动子驱动*α-1, 3GT*基因在C-erb高表达的乳腺癌细胞中靶向表达α-gal表位^[23]^。在前期工作中，课题组成功构建hTERT启动子调控*α-1, 3GT*基因的真核表达载体，实现了*α-1, 3GT*基因在肺癌细胞中靶向合成α-gal。在本研究中，我们发现通过基因导入手段能够使α-1, 3GT持续、稳定地在肿瘤细胞上合成α-gal。然而，hTERT启动子活性相对较弱，如何将有效性与靶向性完美的结合是今后研究的一个方向，将增强子和hTERT启动子联合、CMV/SV40启动子和hTERT启动子联合，或者合成人工hTERT启动子可能是较好的思路之一^[24, 25]^。

总之，本实验通过基因导入手段将异种移植抗原α-gal引入到肿瘤细胞上，观察到了人血清处理后抗体/补体系统的激活，这为肿瘤生物治疗提供了全新的思路，但α-gal诱导的人血清抗肿瘤效果及靶向性问题都需要进一步的研究和探讨。
